# Chemotactic drift speed for bacterial motility pattern with two alternating turning events

**DOI:** 10.1371/journal.pone.0190434

**Published:** 2018-01-19

**Authors:** Evgeniya V. Pankratova, Alena I. Kalyakulina, Mikhail I. Krivonosov, Sergei V. Denisov, Katja M. Taute, Vasily Yu. Zaburdaev

**Affiliations:** 1 Institute of Information Technologies, Mathematics and Mechanics, Lobachevsky State University, Nizhniy Novgorod, Russia; 2 Department of Theoretical Physics, University of Augsburg, Augsburg, Germany; 3 Rowland Institute at Harvard, Harvard University, Cambridge, United States of America; 4 Max Planck Institute for the Physics of Complex Systems, Dresden, Germany; 5 Institute of Supercomputing Technologies, Lobachevsky State University, Nizhniy Novgorod, Russia; University of Heidelberg Medical School, GERMANY

## Abstract

Bacterial chemotaxis is one of the most extensively studied adaptive responses in cells. Many bacteria are able to bias their apparently random motion to produce a drift in the direction of the increasing chemoattractant concentration. It has been recognized that the particular motility pattern employed by moving bacteria has a direct impact on the efficiency of chemotaxis. The linear theory of chemotaxis pioneered by de Gennes allows for calculation of the drift velocity in small gradients for bacteria with basic motility patterns. However, recent experimental data on several bacterial species highlighted the motility pattern where the almost straight runs of cells are interspersed with turning events leading to the reorientation of the cell swimming directions with two distinct angles following in strictly alternating order. In this manuscript we generalize the linear theory of chemotaxis to calculate the chemotactic drift speed for the motility pattern of bacteria with two turning angles. By using the experimental data on motility parameters of *V. alginolyticus* bacteria we can use our theory to relate the efficiency of chemotaxis and the size of bacterial cell body. The results of this work can have a straightforward extension to address most general motility patterns with alternating angles, speeds and durations of runs.

## Introduction

Bacteria are the most numerous living organisms [1]. A variety of shapes, sizes and ways of movement enable them to adapt to different environmental conditions [2, 3]. One of the most common forms of bacteria existence are biofilms, which are multicellular colonies with a complex spatial and metabolic structure forming at interfaces. Ability of individual cells to move and sense environmental signals are crucial for cell aggregation and biofilm formation [4]. Bacteria can move on solid surfaces or swim in liquid media [5]. Although these movements often look like a random motion, bacteria can bias this random motion to move in a certain direction on average. One well-known example of such directed motion is chemotaxis—the ability to alter motility in response to gradients of chemicals [6]. Bacteria in homogeneous environments often exhibit very particular motility patterns, which can greatly affect their ability to perform chemotaxis [7].

One of the most studied motility pattern is “run-and-tumble” of *Escherichia coli* [8]. *E. coli* uses multiple rotating flagella to swim. When all flagella rotate counterclockwise, they form a bundle that drives the bacterium in an approximately straight trajectory of a “run”. When one or more flagella begin to rotate clockwise, the bundle breaks up leading to a change of the swimming direction, known as “tumble” [9]. For *E. coli* the angle between the next and the previous directions is randomly distributed with a mean of approximately 62° [8]. Many bacteria species, in particular those with a single flagellum, completely reverse the direction of their motion after switching of flagellum rotation, thus leading to so called “run-reverse” motility pattern [10, 11].

Importantly, bacteria are able to alternate their motility pattern in response to gradients of certain signaling chemicals. Swimming cells sense the concentration of the signal and extend the duration of the runs, when moving in the direction of the chemical gradient. The chemotactic response of the cells is usually quantified by the average drift velocity in the direction of the gradient. After the key result of de Gennes, who proposed the so called linear theory of chemotaxis, the chemotactic drift speed was calculated for some basic motility patterns of bacteria [7, 12, 13].

Recently advances in bacteria tracking and a careful analysis of bacterial trajectories led to the discovery of more complex motility patterns. For example, a bacterium *V. alginolyticus* exhibits a “run-reverse-flick” pattern with two alternating average turning angles [14–16]. Based on the ensemble measurements it was previously believed that these angles were 180 and 90 degrees [14]. However, new data shows, that while the reversal is indeed universal for all cells, the second turning angle is cell-size dependent [15] and varies significantly between individuals [17]. Importantly previous analytical results for the drift speed of *V. alginolyticus* swimming pattern were obtained under assumption of the second turning (flick) angle of 90°, which dramatically simplifies calculations [7].

In this manuscript, we provide an analytical calculation of the drift speed of chemotactic bacteria moving in a pattern with two alternating arbitrary turning angles. It is thus, to the best of our knowledge, the most general to date extension of the de Gennes result that can be applied to a broad class of bacterial motility patterns. Furthermore it allows us to predict the cell-to-cell variability in the drift speed of *V. alginolyticus* based on published experimental data on the cell-size motility dependence [17].

In the following Section II we outline the derivation of the main result and in Section III combine it with experimental data on *V. alginolyticus* swimming pattern. Section IV is reserved for discussions.

## Chemotactic drift speed calculation for a swimming pattern with two alternating turning events

Various bacteria utilize distinct swimming patterns to navigate their environment. Some of these patterns can be considered as two-step processes (“run-and-tumble” pattern of *E. coli*, for instance), or as four-step processes (as “run-reverse-run-flick”, or “run-reverse-flick” for short, pattern of *V. alginolyticus*) [14]. In the “run-reverse-flick” pattern, a cell swims forward for some time interval and it then backtracks by reversing the direction of the flagellar motor rotation. However, upon resuming forward swimming, the flagellar hook experiences mechanical instability and flicks, causing the cell body to reorient and choose a new swimming direction [18]. Recent experiments show that the average flick angle is cell-size dependent with larger cells having larger flick angles [17] (meaning that larger cells stabilize the flick by counteracting viscous drag force acting on the cell body and their turning angle is closer to reversal). The general theoretical approach presented in this work allows for an analytical treatment of the corresponding chemotactic strategy through the universal de Gennes formalism [13]. We now formulate the model for the bacteria motion with two alternating turning events.

### The pattern of *α* − *β* bacteria motion

Let the swimming pattern of bacteria consist of 4 phases: “Run 1”—movement along a certain direction, “*α*-rotation”—changing the direction of subsequent movement by a random angle Δ*φ*_1_ with a corresponding average cosine value denoted by *α*, *α* ≡ 〈cosΔ*φ*_1_〉, “Run 2”—movement along the new direction, “*β*-rotation”—changing the direction of the subsequent movement by another random turning angle Δ*φ*_2_ with the average cosine denoted by *β*, *β* ≡ 〈cosΔ*φ*_1_〉. The speed of the bacterial movement between two subsequent rotations is considered to be constant and the same for both runs. Interestingly, there are reported cases when the forward and backward swimming speeds are different [10, 19]. Different speeds can also be included in the model, however here we keep them the same to focus on the effect of two alternating angles.

Despite the fact that the times at which the turning events occur are stochastic, the sequence at which the types of turning events follow each other is fixed. Note that, the pattern of *α* − *β* bacteria motion considered in this paper has been observed not only in *V. alginolyticus* but also in other marine bacteria species with a single flagellum [14, 15, 20, 21]. Since it was shown by Altindal et al [22] that in the absence of chemical gradient *V.alginoliticus* demonstrates equal mean values for durations of runs after both turning events and flicks (with *τ* ≈ 0.3s), in our model we assume the running time after both types of reorientation events to be exponentially distributed with the same mean *τ*_*run*_. We should note that the run time distribution measured in experiments often deviates from exponential for short run times. In this respect the exponential distribution is a simplifying assumption, which is, however, crucial for analytical feasibility of the following calculations (for possible affects of non-exponential behavior see [14]). As with two alternating speeds, two different run time distributions can be included without conceptual difficulties but at a cost of lengthier expressions. Durations of both turning events are one order of magnitude shorter than runs and thus usually neglected in the analysis, see however [23]. In our model we treat turnings as instantaneous.

During every run the swimming direction of the cell fluctuates due to the thermal noise in the fluid and possibly due to active processes in the flagellar motor. This effect can be characterized by means of rotational diffusion with a constant *D*_*r*_. The value of *D*_*r*_ can be measured experimentally and in general is in agreement with the estimate of Brownian rotational diffusion of a passive particle of the size of the cell [8]. In general the effect of rotational diffusion is not small; a typical deviation of the swimming direction during a single run can be of the order of 30° [8].

**Fig 1:**
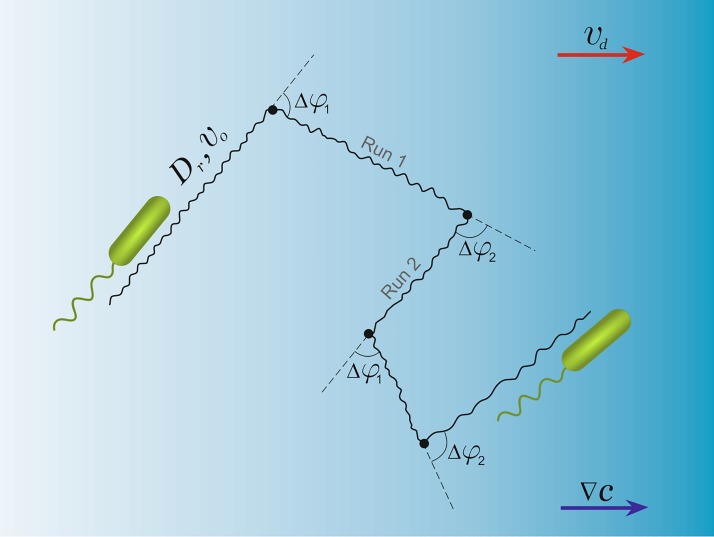
Motility pattern with two turning events. During each run, the speed of the cell *v*_0_ is constant. Motion is nearly straight and is affected by the rotational diffusion *D*_*r*_. The cell changes the direction of its motion during turning events (black dots), where turning angles Δ*φ*_1,2_ are allowed to have two different probability distributions. Importantly, the cell strictly alternates the two types of turning events. When swimming in the gradient of signaling chemicals ∇*c*, the cell can bias its motion and respond with a drift speed *v*_*d*_ in the direction of the gradient, which we want to calculate.

A sketch of the trajectory of *α* − *β* pattern is provided in Fig 1. In the absence of signaling chemicals it is a trajectory of a random walk which can be characterized by a velocity correlation function and the effective long time diffusion constant (see [7] for the corresponding calculations).

### Effect of chemotaxis

In the presence of a chemical gradient bacteria are able to direct their overall random motion towards the attractant. Bacteria possess a chemo-sensory system allowing for temporal integration of the external chemical cues and a delayed response which biases the rotational direction of flagellar motors. When the cell climbs up the gradient it can extend durations of the run phases. The biochemical structure of the chemotaxis response is well understood at least for *E.coli* and many of its features are known experimentally. Here we use the linear model of chemotaxis proposed by de Gennes [13]. This model postulates that the turning frequency of the bacterium is influenced by the experienced concentration of the chemical *c*(*t*) in a following simple form:

$$\begin{array}{c} \lambda \left(t\right)=\lambda_{0}(1-\int_{-\infty }^{t}R\left(t-\tau \right)c\left(\tau \right)d\tau ), \end{array}$$ (1)

where λ_0_ = 1/*τ*_*run*_ and *R*(*t*) is the internal response of the cell, which was measured experimentally for *E. coli* [24]. A typical functional form of the response kernel following from experiments and some analytical arguments on the optimality of the response [25] is:

$$\begin{array}{c} R\left(T\right)=W\lambda_{0}e^{-\lambda_{0}T}\left[1-\frac{\lambda_{0}T}{2}-{\left(\frac{\lambda_{0}T}{2}\right)}^{2}\right], \end{array}$$ (2)

where *W* is a constant characterizing the strength of the response and has the dimension of volume. The central feature of this response kernel is the property

$$\begin{array}{c} \int_{-\infty }^{\infty }R\left(\tau \right)d\tau =0, \end{array}$$ (3)

recognized as the ability of cells to adapt to background concentration of chemicals and sense small gradients even on the high background levels of the signal. The vanishing of the integral means that any background concentration, which is spatially homogeneous, will not affect the tumbling frequency, as seen from Eq (1). Thus, by adopting the response with a zero integral the cell gains the ability to detect small concentration variations independent of the overall constant background concentration of the signaling molecule. Importantly a similar response function was experimentally measured for *V. alginolyticus* bacteria [26]. Although the response for backward and forward motion might differ by a numerical factor of the order of 2 [27], here for simplicity of calculations we assume the same response for both swimming directions. To quantify the effectiveness of chemotaxis we use the so-called chemotactic drift velocity *v*_*d*_ in the concentration of the chemoattractant with a small linear gradient pointing along *Oz* axis:

$$\begin{array}{c} c(z)=|\nabla c|z, \end{array}$$ (4)

where |∇*c*| = const (constant gradient). Without loss of generality we consider a random walk whose first reorientational event at *t* = 0 is an “*α*-rotation”, and, correspondingly, with the turning of type *β* at *t* = *t*_*β*_ > 0. Then we can determine the drift velocity as the sum of average displacements of a run $\langle {\bar{z}}_{\beta }\rangle$ and a subsequent run $\langle {\bar{z}}_{\alpha }\rangle$, divided by mean duration of two runs 2*τ*_*run*_:

$$\begin{array}{c} v_{d}=\frac{\langle {\bar{z}}_{\beta }\rangle +\langle {\bar{z}}_{\alpha }\rangle }{2\tau_{run}}. \end{array}$$ (5)

For $\langle {\bar{z}}_{\alpha }\rangle$ and $\langle {\bar{z}}_{\beta }\rangle$ we calculate the expectation over all possible paths taking into account that the position of the bacterium *z*(*t*) at a time *t* on a particular path is random:

$$\begin{array}{c} \langle {\bar{z}}_{\beta }\rangle =\int_{0}^{\infty }\langle z\left(t\right)p\left(t\right)\rangle dt, \end{array}$$ (6)

where *p*(*t*) is the probability density function for the time corresponding to the run termination event at *t* = *t*_*β*_ on a particular path. Obviously the drift velocity of the bacterial population with *α* = *β* becomes $v_{d}=\langle \bar{z}\rangle /\tau_{run}$.

As was shown by de Gennes, we can first analyze the drift velocity *v*_*δ*_ for a simplified response kernel:

$$\begin{array}{c} R(t)=A\delta (t-T) \end{array}$$ (7)

with a delay time *T* and a strength *A* [12], and then obtain the desired drift speed with the full kernel Eq (2) by a simple integration:

$$\begin{array}{c} v_{d}=\int_{0}^{\infty }R\left(T\right)\frac{v_{\delta }\left(t\right)}{A}dT, \end{array}$$ (8)

where *v*_*δ*_ is the chemotactic drift speed for the delta-response Eq (7). While we give full details of the corresponding derivation in S1 Appendix, here we want to outline the conceptual steps of this calculation.

The derivation of de Gennes [13] relies on the exact answer for the mean run time in case of time dependent turning rate. This is expanded up to the linear order in the gradient of concentration. Finally this gradient can be related to the position of the particle. In both de Gennes’ calculations [13] and previous results on *V. alginolyticus* presence of a 90° turning angle [7] significantly simplified calculations as a turn by 90° completely randomizes new direction and thus erases memory. For a two-step process of *E.coli* with an arbitrary tumble angle and therefore with the non-disappearing memory the problem was solved by Locsei [12]. Our goal is to extend these results to the 4-step pattern. In this case, assuming that the cell has been swimming in the chemical gradient for rather long time, we can estimate the drift velocity via the Eq (5). However, in general (for two alternating turning events with arbitrary angles) the integrals for $\langle {\bar{z}}_{\beta }\rangle$ and $\langle {\bar{z}}_{\alpha }\rangle$ become dependent on each other. This peculiarity is one of the central technical difficulties that should be taken into account. Another important point is the integrand transformation in the Eq (6) alowing to reduce it to an integrable form. Similarly to [7] and [12], to obtain the general expression for the emerged velocity autocorrelation function 〈*v*_*z*_(*t*)*v*_*z*_(*t*′)〉 being valid for any *t* and *t*′, we decomposed it for separate intervals of motion. Taking into account the alternating feature of the considered random walk process, the multipliers in this decomposition can be represented in terms of both *α*- and *β*-type turnings. Integration of the obtained functions gives the expressions for $\langle {\bar{z}}_{\beta }\rangle$ and $\langle {\bar{z}}_{\alpha }\rangle$ whose combination according to Eq (5) leads to the following formal analytical result for the drift velocity in the case of delta-response:

$$\begin{array}{c} v_{\delta }=\frac{v_{0}^{2}}{3}\lambda_{0}A\left|\nabla c\right|[k_{\delta }\cosh(\sqrt{\alpha }\sqrt{\beta }\lambda_{0}T)+m_{\delta }\sinh(\sqrt{\alpha }\sqrt{\beta }\lambda_{0}T)+n_{\delta }] \end{array}$$ (9)

with the coefficients *k*_*δ*_, *m*_*δ*_, *n*_*δ*_:

$$\begin{array}{ll} k_{\delta } & =\frac{\lambda_{0}e^{-\left(2D_{r}+\lambda_{0}\right)T}\left(4D_{r}^{2}\left(2-s_{\alpha \beta }\right)-8D_{r}d_{\alpha \beta }\lambda_{0}-\left(2+s_{\alpha \beta }\right)d_{\alpha \beta }\lambda_{0}^{2}\right)}{2{\left(4D_{r}^{2}+4D_{r}\lambda_{0}-d_{\alpha \beta }\lambda_{0}^{2}\right)}^{2}},\\ m_{\delta } & =\frac{\lambda_{0}e^{-\left(2D_{r}+\lambda_{0}\right)T}\left(4D_{r}^{2}\left(s_{\alpha \beta }-2\alpha \beta \right)-4D_{r}d_{\alpha \beta }\lambda_{0}s_{\alpha \beta }-\lambda_{0}^{2}d_{\alpha \beta }\left(s_{\alpha \beta }+2\alpha \beta \right)\right)}{2{\left(4D_{r}^{2}+4D_{r}\lambda_{0}-d_{\alpha \beta }\lambda_{0}^{2}\right)}^{2}\sqrt{\alpha }\sqrt{\beta }},\\ n_{\delta } & =\frac{\lambda_{0}^{2}\left(4D_{r}^{2}\left(-s_{\alpha \beta }+2\alpha \beta \right)+4D_{r}\lambda_{0}d_{\alpha \beta }s_{\alpha \beta }+\lambda_{0}^{2}d_{\alpha \beta }\left(s_{\alpha \beta }+2\alpha \beta \right)\right)}{2\left(2D_{r}+\lambda_{0}\right){\left(4D_{r}^{2}+4D_{r}\lambda_{0}-d_{\alpha \beta }\lambda_{0}^{2}\right)}^{2}}, \end{array}$$ (10)

where *s*_*αβ*_ = *α* + *β* and *d*_*αβ*_ = −1 + *αβ*. After the integration with the full response kernel Eq (2) the result can be written in a more compact form:

$$\begin{array}{c} \begin{array}{c} v_{d}=\frac{v_{0}^{2}\lambda_{0}^{2}W\left|\nabla c\right|\sum_{j=0}^{7}a_{j}\left(\alpha ,\beta \right)D_{r}^{7-j}\lambda_{0}^{j}}{4\sum_{j=0}^{10}b_{j}\left(\alpha ,\beta \right)D_{r}^{10-j}\lambda_{0}^{j}}, \end{array} \end{array}$$ (11)

where the angle-dependent functions *a*_*j*_(*α*, *β*) and *b*_*j*_(*α*, *β*) can be found in S1 Appendix. Note that, from the orders of polynomials and expressions for non-vanishing coefficients (for any *α* ∈ (−1; 1) and *β* ∈ (−1; 1)) at the highest powers of *D*_*r*_ and λ_0_ follows that the drift velocity is always inversely proportional to the base-line turning rate λ_0_ and to rotational diffusion coefficient in the third degree, i.e. $v_{d}∼1/D_{r}^{3}$. Moreover, it is easy to check, that by setting *β* = 0 we recover the results of [7] and for *α* = *β* the expression of Locsei [12]. Also as in [7] and [12], for any two alternating turning events the drift velocity is quadratic in bacteria speed *v*_0_. This scaling can be qualitatively understood as follows. The length of each run of the cell is proportional to its speed, while the bias in this run is determined by the sensed gradient. During a run, the cell translates the spatial gradient into the temporal concentration gradient where the cell velocity enters as a scaling factor, thus resulting in the overall quadratic dependence of the drift speed on cell velocity. Before applying the general result of Eq (11) to experimental data we first explore its dependence on parameters and compare to the results of numerical simulations.

### Verification of analytically obtained formula for the drift speed by numerical simulations

The drift speed is linearly proportional to the gradient of concentration |∇*c*| and to the amplitude of the response *W*. Dependence on rotational diffusion constant and the turning frequency (without the gradient) is more involved. Our main goal is to check the angular dependence of the result with numerica experiment.

Due to the specific form of the response function, Eq (2), we can benefit from the so-called “embedding” technique [28, 29]. We transform Eq (1), which is nonlocal in time, into a system of three linear differential equations corresponding to the number of terms in the response function and that are now local in time. Bacteria motility is modeled as a sequence of short steps, during which the bacteria performs a motion by integrating its velocity in time and integrates the chemical concentration of the attractant in space. The velocity vector performs an unbiased rotational diffusion. Every step is completed with a “coin flipping”, which decides whether the bacteria should perform a re-orientation or not. With decreasing of the time step Δ*t*, the discrete process converges and starts to reproduce the continuous stochastic dynamics described in Section II. A more detailed description of the numerical algorithm is given in S2 Appendix.

We model the motility of an ensembel of *N* ≈ 0.5 ⋅ 10^6^ cells during the time interval *T* = 2000 s ≈ 33 min with the time step Δ*t* = 0.01s were performed for the following fixed parameters: *v*_0_ = 45 *μ*ms^−1^ and *τ*_*run*_ = 0.3 s [14]. The value of the rotational diffusion constant *D*_*r*_, as well as parameters *α* and *β*, were varied. We also performed simulations for different strengths of the linear gradient.

**Fig 2:**
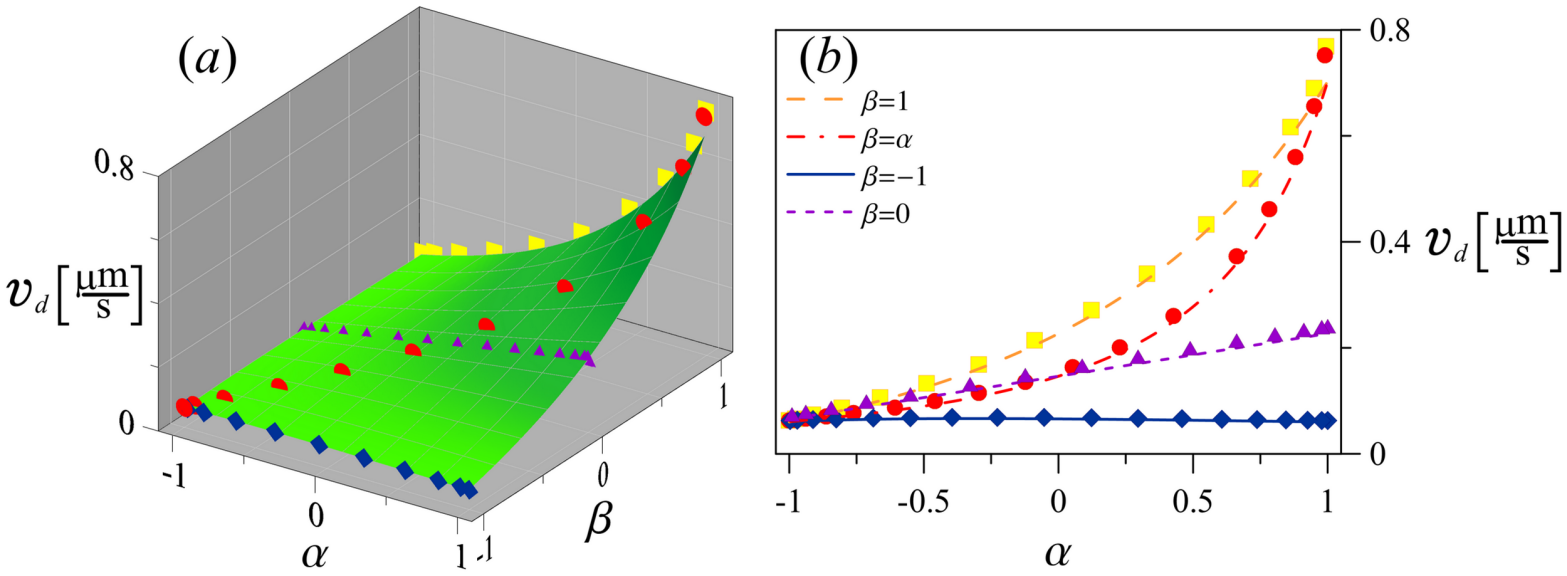
Drift speed without rotational diffusion. (a) Analytically obtained function *v*_*d*_(*α*, *β*), see Eq (11) is shown as a green surface and numerically obtained results as symbols for *D*_*r*_ = 0.0 rad^2^s^−1^, |∇*c*| = 0.05 *μm*^−4^ and *W* = 0.0458 *μ*m^3^. (b) Comparison of analytically (lines) and numerically (symbols) obtained drift speed dependences on the parameter *α* for four values of *β*: *β* = 1.0 (yellow), *β* = *α* (red), *β* = −1.0 (blue), *β* = 0.0 (purple).

**Fig 3:**
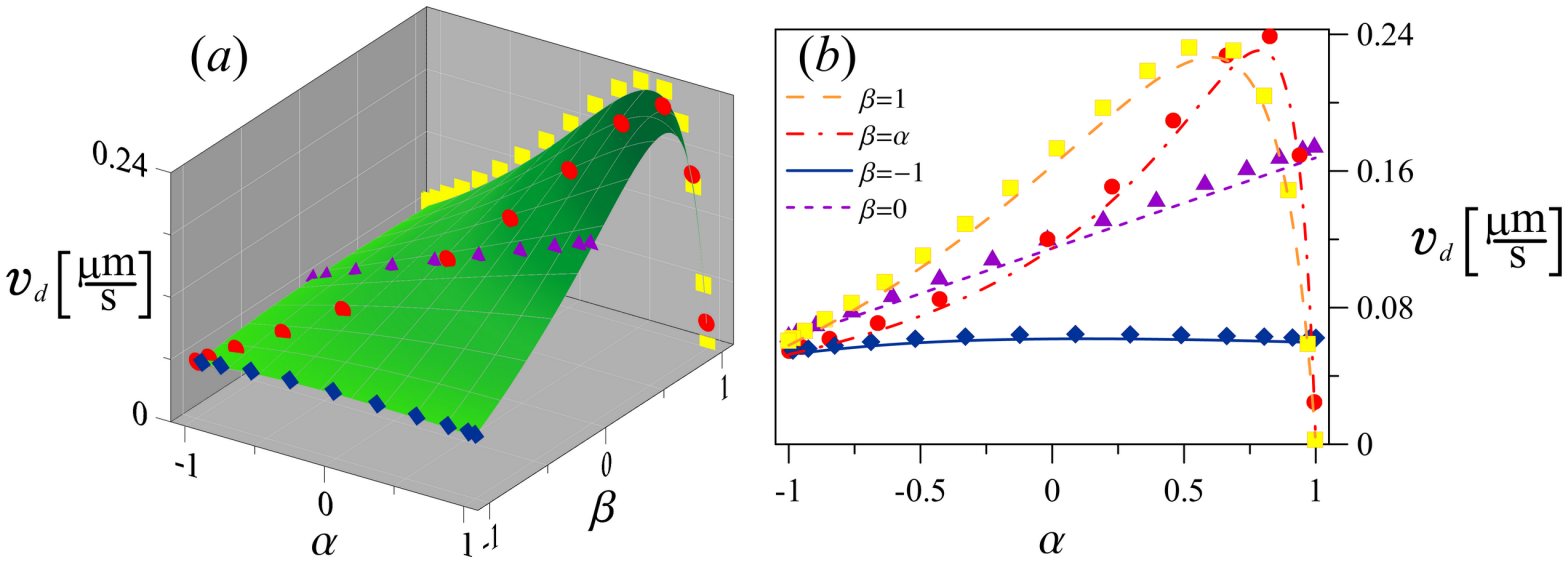
Drift speed with rotational diffusion. (a) Analytically obtained function *v*_*d*_(*α*, *β*), shown as surface, and numerically obtained result as points for *D*_*r*_ = 0.2 rad^2^s^−1^, |∇*c*| = 0.05 *μm*^−4^ and *W* = 0.0458 *μ*m^3^. (b) Comparison of analytically (lines) and numerically (symbols) obtained drift speed dependences on the parameter *α* for four values of *β*: *β* = 1.0 (yellow), *β* = *α* (red), *β* = −1.0 (blue), *β* = 0.0 (purple).

For *D*_*r*_ = 0 rad^2^s^−1^, analytically obtained drift velocity in the general case of two alternating turning events is presented as a green surface in Fig 2. Symbols show the numerically calculated values of the drift speed for three particular types of the swimming patterns: *β* = 1.0 (yellow), *β* = *α* (red), *β* = −1.0 (blue), *β* = 0.0 (purple). We see that as expected *v*_*d*_(*α*, *β*) is symmetric with respect to the plane *α* = *β*. The drift speed is increasing when both *α* and *β* approach 1. However when *α* = *β* = 1 without rotational diffusion we have directed ballistic motion and *v*_*d*_ = 0. Agreement is similarly good for the case *D*_*r*_ = 0.2 rad^2^s^−1^, see results on Fig 3. We see that the rotational diffusion reduces the drift velocity and also leads to the appearance of a maximum along the line *α* = *β* with the drift velocity smoothly going to zero as *α* and *β* approach 1.

**Fig 4:**
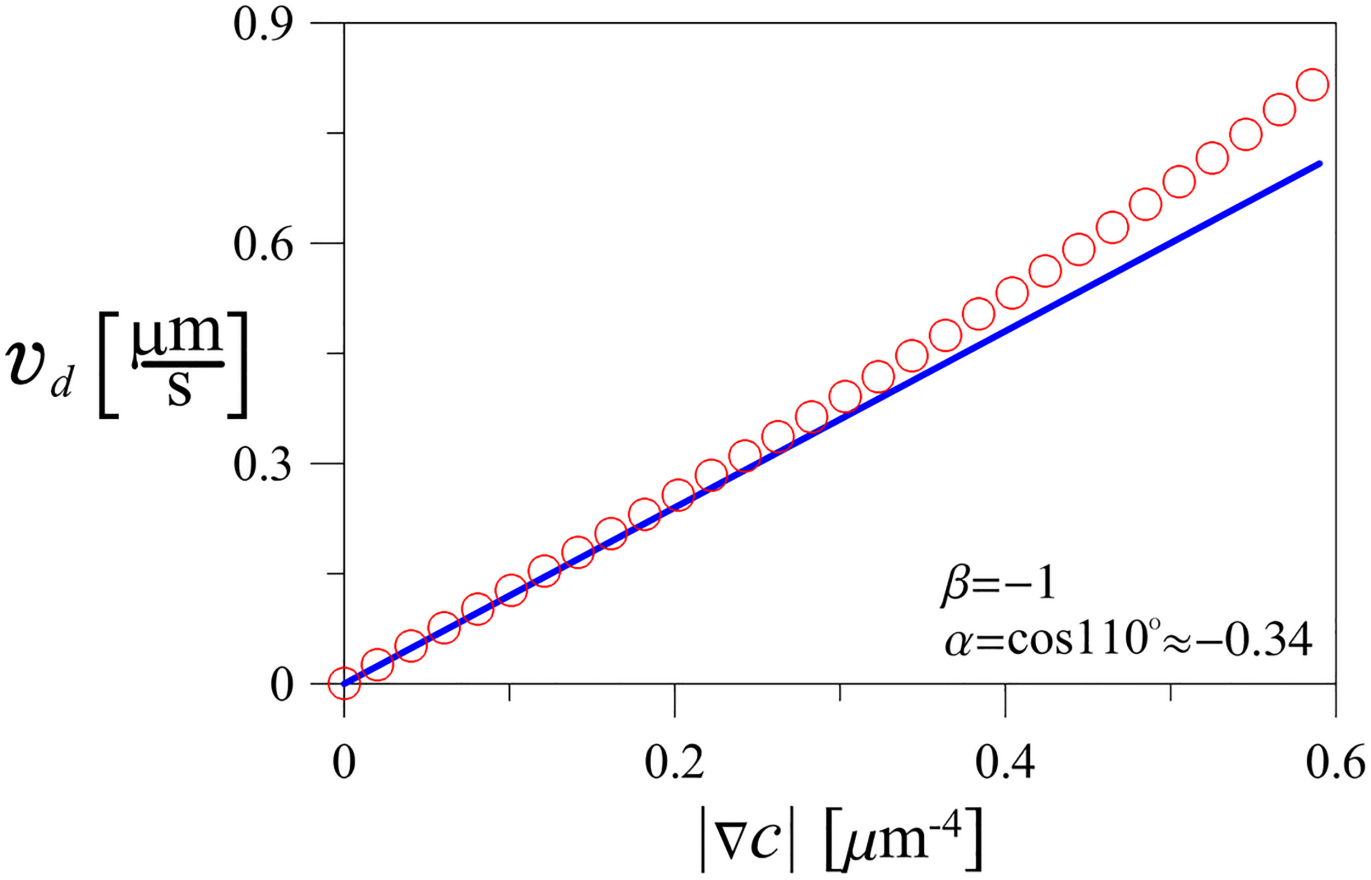
Drift speed as a function of the chemoattractant gradient strength. Analytically obtained predictions (curve) agree with numerical results (symbols) up to the gradient values of ⋍ 0.5 *μm*^−4^ (*D*_*r*_ = 0.2 rad^2^s^−1^, λ = 3.3 s^−1^, *α* ≈ −0.34, *β* = −1).

Numerical experiments are consistent with analytical calculations within 10% error up to the gradient value |∇*c*| ≈ 0.6 *μm*^−4^, Fig 4. This once again highlights the fact, that here we used a linear theory of chemotaxis, which relies on the expansion with respect to a small gradient. Now we can apply our analytical results to the experimental data of *V. alginolyticus* motility.

## Chemotactic drift speed estimation based on experimental data for *V. alginolyticus* swimming pattern

**Fig 5:**
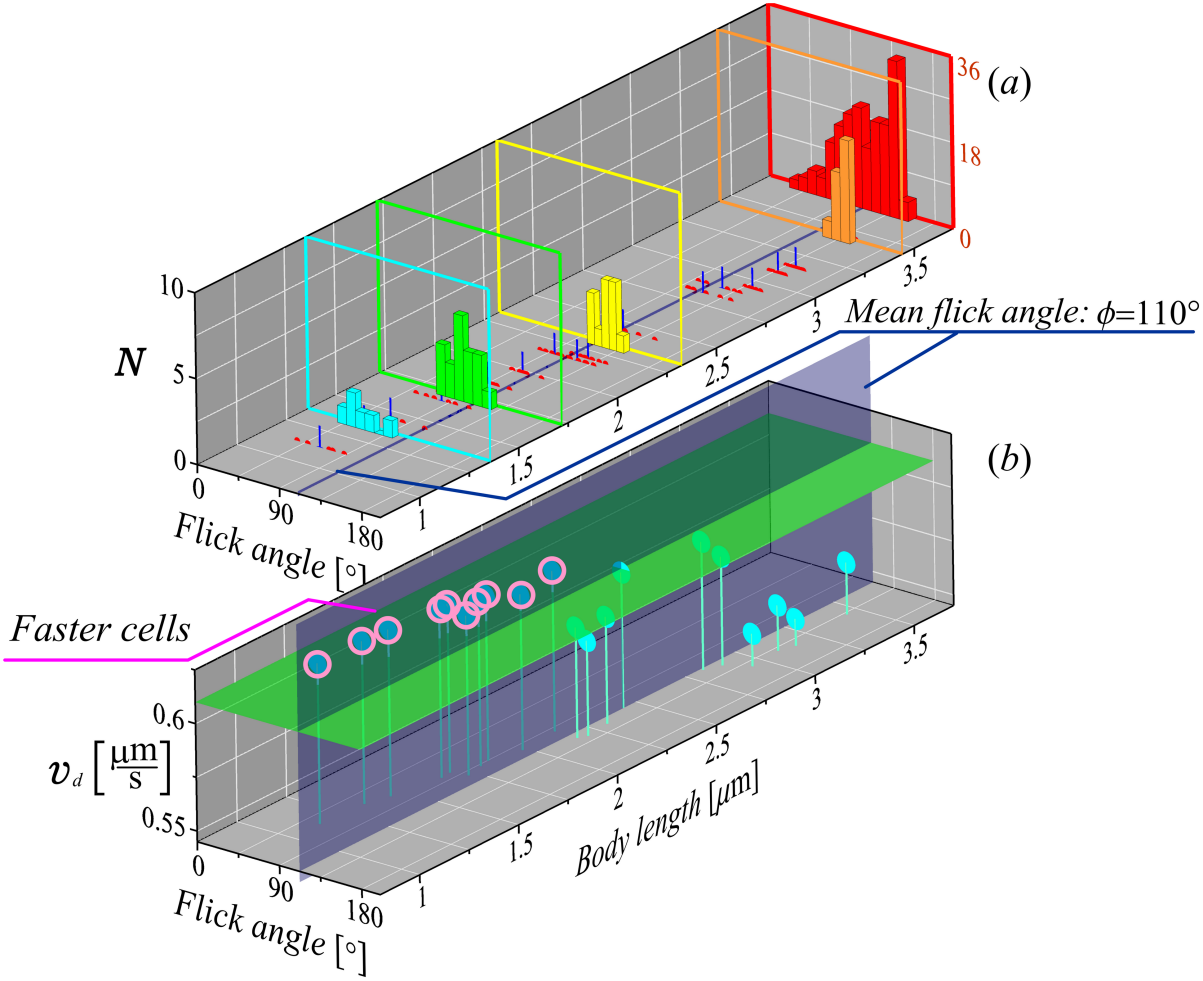
Variability of the drift velocity with the cell body size. (a) Histograms showing the distribution of flick angles with various body lengths (data from [17]). Red histogram represents all measured flick angles—170 events for 20 cells with various body lengths. Flick angles for four particular values of cellular body lengths are shown by narrow color histograms (*l* = 3.45 *μ*m, orange; *l* = 2.31 *μ*m, yellow; *l* = 1.72 *μ*m, green; *l* = 1.35 *μ*m, cyan histogram). The data obtained for 20 individuals (each of which is displayed at least 6 flicks in their trajectory) are shown as red points. Small blue ticks show the mean flick angles obtained from individual distributions. Black tick labels of the vertical axis correspond to the histograms of individual cells, whereas the red tick labels correspond to the red histogram for all cells. (b) Analytically obtained drift speed calculated for the mean flick angle of the considered cells, as a function of the angle and the corresponding body length. For results the following parameters were used: |∇*c*| = 0.5 *μ*m^−4^, λ = 3.3 s^−1^, *v*_0_ = 45 *μ*ms^−1^, *D*_*r*_ = 0.2 rad^2^s^−1^, *W* = 0.0458 *μ*m^3^ and *β* = cos 172°.

Recently, the trajectories obtained by the high-throughput 3D bacterial tracking method [17] revealed some interesting details about the “run-reverse-flick” swimming pattern of the marine bacterium *V. alginolyticus*. The ability to capture individual trajectories that contain a sufficient number of flicks and the information about the size of the bacteria provided new insights into inter-individual variability. In Fig 5a, the experimentally measured distribution of the flick angles is shown as a red histogram. It is rather broad and has one well pronounced maximum. In similar previous measurements [14], the flick angles were reported to be randomly distributed with an average turning angle close to 90°.

Importantly, comparing the measured angles for the cells with different sizes revealed that individuals actually show very narrow flick angle distributions with different means [orange, yellow, green and cyan histograms in Fig 5a]. Moreover, there is a correlation between the cells-body length and the angle between the forward and reverse runs during the flick, i.e. between the cell’s size and *α*. Reorientation during a flick is counteracted by the viscous drag [15] and thus larger cells have a flick angle closer to 180° [17].

As was shown in [17], the mean value of the reversal angle for the bacteria in the considered population was 172°. In this case, the reversal has a narrow distribution and almost does not change from cell to cell.

Therefore, given the more detailed data of [17], simplified “run-reverse-flick” motility models using the previously measured mean flick angle of ∼90° [14] and assuming a completely random swimming direction during flicks, should be reconsidered. In this section, we will use the detailed data of [17] to calculate the drift velocity from our analytical results and show how this velocity varies with the cell size. Specifically, by substituting into Eq (11) the experimentally measured values of the mean cosines of both turning events, i.e. *β* = cos 172° ≈ −0.99 for the reversal for all cells and size-dependent *α*, the drift velocities can be calculated for various sizes of cells. This shows that the bacteria having various body lengths and, consequently, various mean flicking angles, demonstrate noticeable variability. The drift velocity obtained for bacteria having small size (up to 2 *μm*) are circled in Fig 5b. This shows that the smaller cells have higher drift speed and, therefore, can reach a chemoattractant source faster than the others, and the difference between slowest and fastest cells is of the order of 10%. For results in Fig 5b we used the following parameters: |∇*c*| = 0.5 *μ*m^−4^, λ = 3.3 s^−1^, *v*_0_ = 45 *μ*ms^−1^, *D*_*r*_ = 0.2 rad^2^s^−1^, *W* = 0.0458 *μ*m^3^ and *β* = cos 172°. It is important to note, that the value of *W* we borrowed from *E. coli* bacteria. For *V. alginolyticus* it can be easily back-calculated from the experimentally measured drift speed in a small gradient (as it was done before for *E. coli* [7]). However, to the best of our knowledge, the drift speed of *V. alginolyticus* in a small linear gradient was not measured before. It would be an important next step, also allowing for the experimental validation of our theoretical predictions.

## Discussion

Continuously advancing measurements techniques allow us to get a more detailed information on a bacterial behavior at the level of individual cells. We are at the point when the variability between cells can and should be accounted for in our quantitative analysis of motility and chemotaxis. In this work we considered one of the most general swimming patterns containing two alternating turning angles. This analytical framework allows for a straightforward analysis of *V. alginolyticus* cells with their intrinsic variability: cells of different sizes have different flick angles and thus can be naturally accommodated by the model. The conceptual challenge of the provided calculation of the chemotactic drift velocity is in the non-disappearing memory during the turning events.

**Fig 6:**
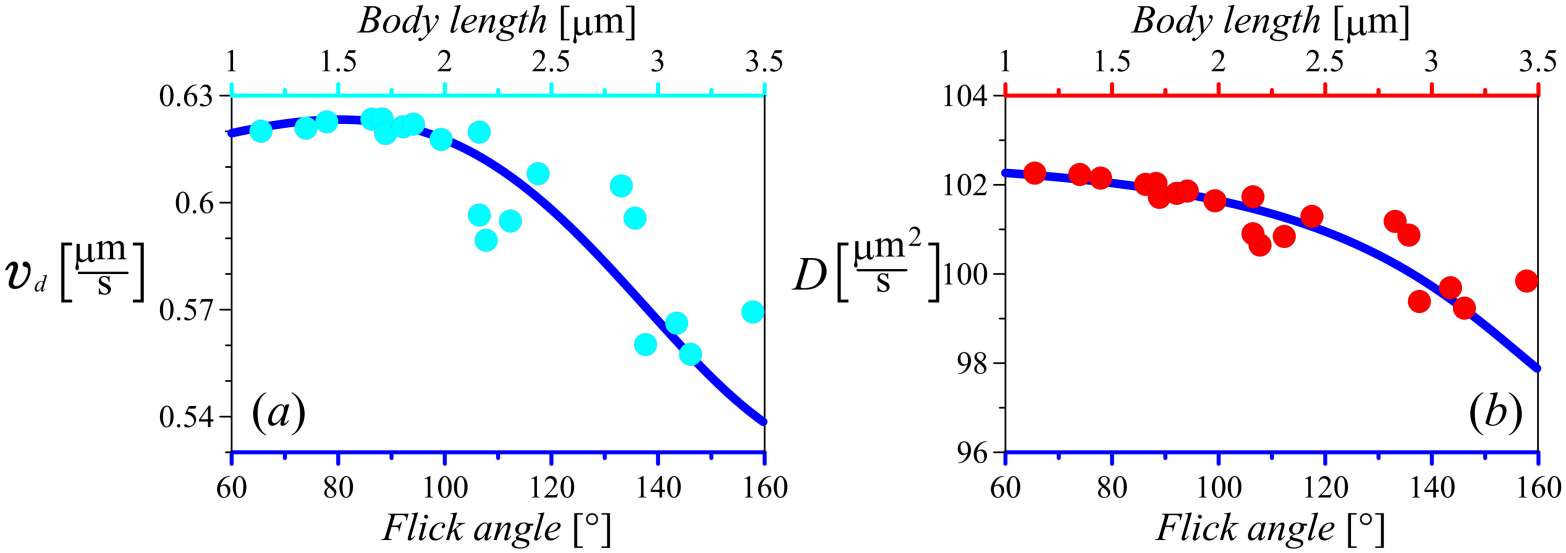
Analytically obtained drift speed and diffusion constant as functions of the mean flick angle and the corresponding body length. The curves represent the angle-dependent characteristics obtained from Eqs (11) and (12). To plot the drift speed and the diffusion constant as a function of cell size, we use the data of [17] to relate the size to the turning angle, and use that angle in analytical results Eqs (11) and (12) (these values are shown by symbols). The fact that data based on cell sizes line up with the theoretical curves as functions of angles indicates an approximately linear relation between the cell size and the cosine of the turning angle, which is in agreement with data presented in [17]. The parameters are λ = 3.3 s^−1^, *v*_0_ = 45 *μ*ms^−1^, *D*_*r*_ = 0.2 rad^2^s^−1^ and *β* = cos 172°.

Although we observe a noticeable difference in the drift speeds of bacteria of different sizes, for the considered small gradients this difference is of the order of 10%, see Fig 6a. We should note that although there is a maximum of the drift speed at a certain flick angle, which depends on the parameters of the swimming pattern in a nontrivial way, this maximum is not very pronounced. Thus bacteria with a rather broad range of flick angles have comparable drift velocities. Interestingly, a much stronger dependence on the second turning angle was recently reported for *S. putrefaciens* bacterium [30]. This effect has a natural explanation. In *S. putrefaciens* the duration of the run after reversal and prior to flick is much shorter than that of the run after the flick. Thus the motility pattern is effectively similar to the *E. coli* run and tumble with a single turning angle. In that case the effect of the angle on the drift velocity is very pronounced (see Fig 3b red curve). Importantly, further customization of the model is possible. We can consider different (but limited to exponential) distributions for two run times, different forward and backward speeds, and even different memory kernels. Thus the example of *S. putrefaciens* can be also put on the analytical footing developed in this paper.

Another important parameter affecting the drift speed is the rotational diffusion (cf. Figs 2 and 3). Potentially, rotational diffusion is another parameter that can depend on the cell size and it would be important to check this effect both theoretically and experimentally in the future.

For bacterial chemotaxis it is not only important how fast cells can move towards the higher concentration of signaling chemicals, but also how well can they localize themselves near the source of the gradient. Thus not only the drift velocity but also the effective diffusion constant of the bacterial motility pattern play an important role [31]. One can quantify the localization ability by considering for example the ratio *v*_*d*_/*D*, which describes the competition between the drift and diffusive spreading and has the meaning of the inverse characteristic length. The diffusion constant of the motility pattern with two arbitrary turning angles was calculated in [7]:

$$\begin{array}{c} D=\frac{v_{0}^{2}}{6}\frac{\left(2+\alpha +\beta \right)\lambda +4D_{r}}{\left(1-\alpha \beta \right)\lambda^{2}+4D_{r}\left(\lambda +D_{r}\right)}. \end{array}$$ (12)

As for the drift velocity, substituting into Eq (12) the mean cosines of alternating size-dependent flicks *α* and size-independent reversal angles *β*, one can estimate the impact of bacterial body length in cell’s localization ability near the source of attracting chemicals. It is easy to see that in general there is a strong dependence of the diffusion constant on the turning angles. However, if one of the angles is fixed in the reversal mode, as in the case of *V.alginolyticus*, the variation of the flick angle does not lead to large changes in the effective diffusion constant, see Fig 6b (for a smaller rotational diffusion constant the effect of the flick angle would be more pronounced). Note that, as for the drift velocity, the diffusion coefficient is also quadratic in bacteria speed *v*_0_. This scaling is due to a simple random walk estimate of the diffusion constant as the mean squared step distance (*v*_0_*τ*_*run*_)^2^ divided by the mean run time *τ*_*run*_.

With this information at hand we have a full theoretical tool set to quantify bacterial chemotaxis in the small gradient approximation. The theoretical results presented here for a 4-step pattern, allow us to predict the drift as a function of the cell size. Importantly, these predictions could be verified experimentally where the drift speeds of cells along the linear gradient can be correlated with their size. One of the possible applications of the size-dependent drift velocity is in cell sorting, where after a certain time of motion along the linear gradient the cells of different sizes would be found at distinct positions corresponding to their drift speed.

We think that the analytical relations, such as chemotactic drift speed considered here, provide a rigorous link between motility pattern and chemotactic response and thus can be used in experiments to infer the yet unknown parameters of bacterial sensitizing based on tracking or chemotactic drift experiments.

## Supporting information

S1 Appendix Drift speed calculation. (PDF)

S2 Appendix Simulation algorithm. (PDF)
