# Supplementary material for: Chemotactic drift speed for bacterial motility pattern with two alternating turning events
Source: PLoS One. 2018 Jan 19;13(1):e0190434. doi: 10.1371/journal.pone.0190434 (PMC5774696; doi:10.1371/journal.pone.0190434)
Supplement: S1 Appendix — (PDF) [file pone.0190434.s001.pdf]

Chemotactic drift speed for bacterial motility pattern with two alternating turning events

Evgeniya V. Pankratova[1*], Alena I. Kalyakulina[1], Mikhail I. Krivonosov[1], Sergei V. Denisov[1, 2], Katja M. Taute[3], Vasily Yu. Zaburdaev[4, 5].

1 Institute of Information Technologies, Mathematics and Mechanics, Lobachevsky State University, Nizhniy Novgorod, Russia
2 Department of Theoretical Physics, University of Augsburg, Germany
3 Rowland Institute at Harvard, Harvard University, Cambridge, USA
4 Max Planck Institute for the Physics of Complex Systems, Dresden, Germany
5 Institute of Supercomputing Technologies, Lobachevsky State University, Nizhniy Novgorod, Russia

*E-mail: evgenia.pankratova@itmm.unn.ru

Supporting information

S1 Appendix. Drift speed calculation

The motility patterns of bacteria can be considered as a process composed of two main alternating phases of motion: "runs" and "turnings". Although the angular changes during the turning events are stochastic, analysis of the experimental data shows that cells are able to exhibit directional persistence that can be different for various species. *E. coli* cells have a motility pattern with a preferential turning angle $\Delta\varphi$ that can be characterized by the parameter $\alpha = \langle \cos \Delta\varphi \rangle$. *V. alginolyticus* bacteria perform motility with two alternating re-orientation events that can be specified by two parameters $\alpha = \langle \cos \Delta\varphi_1 \rangle$ and $\beta = \langle \cos \Delta\varphi_2 \rangle$.

The calculation of chemotactic drift speed v_d for motility with a single directional parameter was performed by Locsei [1]. Here, we extend his approach to more complicated case of bacterial chemotaxis with two alternating turning events. Note that, the algorithm presented below can be easily extended for random walks with arbitrary number of alternating turning events. Moreover, taking into account the change of parameters (mean run durations and rotational diffusion coefficients) between appointed turnings, more accurate prediction for the chemotactic drift speed can be obtained. Here, for simplicity, we restrict our analysis by assumption that all parameters for "run"-phases of the considered pattern are the same.

Without loss of generality we consider a random walk whose first turning event for $t = t_\beta > 0$ is specified by the parameter β, i.e. with a turning of type α at $t = 0$.

Let z_β be the z location of a cell at the end of a run, relative to its position at the beginning of the considered random walk. At $t = t_\beta$, the direction of motion is changed with parameter β and the cell performs a run of duration t_α. To obtain the drift velocity v_d in a first-order expansion with respect to $|\nabla c|$, we determine the average displacement of a run $\langle \overline{z}_\beta \rangle$ and a subsequent run $\langle \overline{z}_\alpha \rangle$. In our calculations, as in [1], we treat the duration of turning events as negligible. As the expressions for $\langle \overline{z}_\beta \rangle$ and $\langle \overline{z}_\alpha \rangle$ are first order in $|\nabla c|$, the mean duration of "two runs" process is given by $2\tau_{run}$, and the chemotaxis drift speed becomes

$$v_d = \frac{\langle \overline{z}_\beta \rangle + \langle \overline{z}_\alpha \rangle}{2\tau_{run}}. \quad \text{(S1)}$$

For $\langle \overline{z}_\alpha \rangle$ and $\langle \overline{z}_\beta \rangle$ we calculate expectation over all possible paths taking into account that the position of the bacterium at a time t on a particular path is random.

In the first step, we calculate the mean displacement of the run $\langle \overline{z}_\beta \rangle$:

$$\langle \overline{z}_\beta \rangle = \langle \int_0^\infty z(t)p(t)dt \rangle. \tag{S2}$$

where $z(t)$ is position of a cell at time t in particular path, and $p(t)$ is the probability density, that a run starts at $t = 0$ and stops and $t = t_\beta$. Since the paths are independent of t_β, we may take the path expectation inside the integral over run times

$$\langle \overline{z}_\beta \rangle = \int_0^\infty \langle z(t)p(t) \rangle dt, \tag{S3}$$

where $p(t)$ is given by [1]

$$p(t) = \lambda(t) e^{-\int_0^t \lambda(t')dt'}. \tag{S4}$$

In accordance with the idea of de Gennes we assume that the turning rate $\lambda(t)$ in (S4) in the presence of chemoattractant becomes

$$\lambda(t) = \lambda_0 [1 - \Delta(t)], \tag{S5}$$

where $\lambda_0 = 1/\tau_{run}$ is the mean turning rate of bacteria, and the fractional change in $\lambda(t)$ caused by the presence of chemicals in the environment, is:

$$\Delta(t) = \int_{-\infty}^t c(t')R(t-t')dt'. \tag{S6}$$

In (S6) $c(t')$ is chemoattractant concentration experienced by the cell at time t' and $R(t)$ is the cell's response function. In the case of small chemical gradient $|\nabla c|$ in z direction, the chemoattractant concentration $c(t)$ at the cell's position $z(t)$ can be written in the form

$$c(t) = c_0 + |\nabla c| z(t). \tag{S7}$$

Substituting (S7) into (S6) yields

$$\Delta(t) = \int_{-\infty}^t [c_0 + |\nabla c| z(t')]R(t-t')dt' = c_0 \int_{-\infty}^t R(t-t')dt' + |\nabla c| \int_{-\infty}^t z(t')R(t-t')dt'. \tag{S8}$$

Since the response function R has a zero integral, an additive constant in (S8) has no effect on $\Delta(t)$. Therefore, the equality (S8) can be rewritten in the form

$$\Delta(t) = |\nabla c| \int_{-\infty}^t z(t')R(t-t')dt'. \tag{S9}$$

To simplify forthcoming calculations, we first consider the special case of a delta-response in time

$$R(t) = A\delta(t-T) \tag{S10}$$

with delay time T and strength A, as it was done in [2]. In this case, the fractional change in turning rate becomes

$$\Delta(t) = A|\nabla c| \int_{-\infty}^t z(t')\delta(t-T-t')dt' = A|\nabla c| z(t-T). \tag{S11}$$

Thus, taking into account that for the run lasting from $t = 0$ until $t = t_\beta$, the average displacement (S3) after integration by parts can be written as

$$\langle \overline{z}_{\beta} \rangle = \int_0^{\infty} \langle v_z(t) e^{-\int_0^t \lambda(t')dt'} \rangle dt, \tag{S12}$$

where $v_z(t) = dz(t)/dt$ and $\lambda(t') = \lambda_0 [1 - A|\nabla c| z(t' - T)]$, one can obtain

$$\langle \overline{z}_{\beta} \rangle = \int_0^{\infty} \langle v_z(t) e^{-\lambda_0 t} e^{\lambda_0 A|\nabla c| \int_0^t z(t'-T)dt'} \rangle dt. \tag{S13}$$

For a small chemical gradient ($\Delta(t) \ll 1$) expanding the exponential and keeping only the first-order terms in $|\nabla c|$, i.e. $e^{\lambda_0 A|\nabla c| \int_0^t z(t'-T)dt'} \approx 1 + \lambda_0 A|\nabla c| \int_0^t z(t'-T)dt'$, one obtains

$$\langle \overline{z}_{\beta} \rangle = \int_0^{\infty} \langle v_z(t) \rangle e^{-\lambda_0 t} dt + \lambda_0 A|\nabla c| \int_0^{\infty} e^{-\lambda_0 t} \left[\int_0^t \langle z(t'-T) v_z(t) \rangle dt' \right] dt. \tag{S14}$$

Substitution of $\langle v_z(t) \rangle = e^{-2D_r t} \langle v(0^+) \rangle$ [1] into the first integral of (S14) and using $z(t) = \int_0^t v_z(s)ds$ for the second integral of (S14) yields

$$\langle \overline{z}_{\beta} \rangle = \frac{\langle v(0^+) \rangle}{\lambda_0 + 2D_r} + \lambda_0 A|\nabla c| \int_0^{\infty} e^{-\lambda_0 t} \left[\int_0^t \left(\int_0^{t'-T} \langle v_z(s) v_z(t) \rangle ds \right) dt' \right] dt. \tag{S15}$$

Also within the first order approximation, the velocity is governed by an isotropic distribution, which implies

$$\langle v_z(s) v_z(t_{\beta}) \rangle = \frac{v_0^2}{3} \langle \mathbf{e}(s) \cdot \mathbf{e}(t_{\beta}) \rangle \tag{S16}$$

where v_0 is a constant speed during a run, and the directional correlation function reads [3]

$$\langle \mathbf{e}(s) \cdot \mathbf{e}(t_{\beta}) \rangle = \begin{cases} e^{-2D_r(t_{\beta}-s)} & \text{if } 0 \le s < t_{\beta}, \\ \alpha e^{-2D_r t_{\beta}} e^{-(\lambda_0+2D_r)|s|} \cdot & \\ \left[\sqrt{\frac{\beta}{\alpha}} \sinh\left(\sqrt{\alpha}\sqrt{\beta}\lambda_0|s|\right) + \cosh\left(\sqrt{\alpha}\sqrt{\beta}\lambda_0|s|\right) \right] & \text{if } s < 0. \end{cases} \tag{S17}$$

To obtain the second line of (S17), we made a decomposition

$$\langle \mathbf{e}(s) \cdot \mathbf{e}(t_{\beta}) \rangle = \langle \mathbf{e}(s) \cdot \mathbf{e}(0^-) \rangle \langle \mathbf{e}(0^-) \cdot \mathbf{e}(0^+) \rangle \langle \mathbf{e}(0^+) \cdot \mathbf{e}(t_{\beta}) \rangle, \tag{S18}$$

where the direction correlation function for directions immediately before and after the turning at $t = 0$ equals to α due to our choice of the considered motility pattern:

$$\langle \mathbf{e}(0^-) \cdot \mathbf{e}(0^+) \rangle = \alpha, \tag{S19}$$

whereas the last multiplier in (S18) can be obtained from the Fokker-Planck equation (see S2 Appendix and [1] for details).

$$\langle \mathbf{e}(0^+) \cdot \mathbf{e}(t_{\beta}) \rangle = e^{-2D_r t_{\beta}}, \tag{S20}$$

where D_r is the rotational diffusion coefficient describing the influence of rotational Brownian motion during the "run"-phase of bacterial swimming. To determine the first multiplier in the product (S18) we should take into account that for $t < 0$ the cell's motility obeys to the pattern with two alternating turning events with additional

reorientations due to rotational Brownian motion during the runs. For this case, the direction correlation function for the process, whose first run interrupts by a turning of type β becomes

$$\langle \mathbf{e}(s) \cdot \mathbf{e}(0^-) \rangle = e^{-(\lambda_0 + 2D_r)|s|} \left[\sqrt{\frac{\beta}{\alpha}} \sinh\left(\sqrt{\alpha}\sqrt{\beta}\lambda_0 |s|\right) + \cosh\left(\sqrt{\alpha}\sqrt{\beta}\lambda_0 |s|\right) \right], \tag{S21}$$

see [3] for details. After inserting (S16) and (S17) into the second integral of (S15) for the mean displacement of the first run we obtain

$$\langle \overline{z}_\beta \rangle = \frac{\langle v(0^+) \rangle}{\lambda_0 + 2D_r} + \frac{v_0^2}{3} \lambda_0 A |\nabla c| \left[k_\beta \cosh\left(\sqrt{\alpha}\sqrt{\beta}\lambda_0 T\right) + m_\beta \sinh\left(\sqrt{\alpha}\sqrt{\beta}\lambda_0 T\right) + n_\beta \right], \tag{S22}$$

where

$$\begin{aligned} k_\beta &= \frac{\lambda_0 e^{-(2D_r + \lambda_0)T}(2D_r + \lambda_0 + \alpha\lambda_0)}{(2D_r + \lambda_0)(4D_r^2 + 4D_r\lambda_0 + (1 - \alpha\beta)\lambda_0^2)}, \\ m_\beta &= \frac{\lambda_0 e^{-(2D_r + \lambda_0)T}\sqrt{\alpha}(2D_r + \lambda_0 + \beta\lambda_0)}{\sqrt{\beta}(2D_r + \lambda_0)(4D_r^2 + 4D_r\lambda_0 + (1 - \alpha\beta)\lambda_0^2)}, \\ n_\beta &= \frac{-\lambda_0^2\alpha(2D_r + \lambda_0 + \beta\lambda_0)}{(2D_r + \lambda_0)^2(4D_r^2 + 4D_r\lambda_0 + (1 - \alpha\beta)\lambda_0^2)}. \end{aligned} \tag{S23}$$

Proceeding in the same way as for z_β, one can obtain the mean displacement of the second run, which is interrupted by turning of type α:

$$\langle \overline{z}_\alpha \rangle = \frac{\langle v(t_\beta^+) \rangle}{\lambda_0 + 2D_r} + \frac{v_0^2}{3} \lambda_0 A |\nabla c| \left[k_\alpha \cosh\left(\sqrt{\alpha}\sqrt{\beta}\lambda_0 T\right) + m_\alpha \sinh\left(\sqrt{\alpha}\sqrt{\beta}\lambda_0 T\right) + n_\alpha \right], \tag{S24}$$

where

$$\begin{aligned} k_\alpha &= \frac{\lambda_0 e^{-(2D_r + \lambda_0)T}(2D_r + \lambda_0 + \beta\lambda_0)}{(2D_r + \lambda_0)(4D_r^2 + 4D_r\lambda_0 + (1 - \alpha\beta)\lambda_0^2)}, \\ m_\alpha &= \frac{\lambda_0 e^{-(2D_r + \lambda_0)T}\sqrt{\beta}(2D_r + \lambda_0 + \alpha\lambda_0)}{\sqrt{\alpha}(2D_r + \lambda_0)(4D_r^2 + 4D_r\lambda_0 + (1 - \alpha\beta)\lambda_0^2)}, \\ n_\alpha &= \frac{-\lambda_0^2\beta(2D_r + \lambda_0 + \alpha\lambda_0)}{(2D_r + \lambda_0)^2(4D_r^2 + 4D_r\lambda_0 + (1 - \alpha\beta)\lambda_0^2)} \end{aligned} \tag{S25}$$

and $v(t_\beta^+)$ is the z component of the cell's velocity at the beginning of the second run. As before, to obtain the directional correlation function for $t < t_\alpha$, we should expand $\langle \mathbf{e}(s) \cdot \mathbf{e}(t_\alpha) \rangle$ into a product of direction correlations between different times. However, since we consider the motility with regularly alternating reorientations of bacteria, we can claim its similarity to the previous one (S17) with obvious replacement for the following parameters $t_\beta \to t_\alpha$ and $\alpha \leftrightarrow \beta$.

Having found the integrals in expressions for $\langle \overline{z}_\beta \rangle$ and $\langle \overline{z}_\alpha \rangle$, we turn our attention to $\langle v(0^+) \rangle$ and $\langle v(t_\beta^+) \rangle$. In our case, for random walk with a turning of type α at $t = 0$ and type β at $t = t_\beta$, using the definitions for the parameters α and β we can write

$$\begin{aligned} \langle \mathbf{e}(0^-) \cdot \mathbf{e}(0^+) \rangle &= \alpha, \\ \langle \mathbf{e}(t_\beta^-) \cdot \mathbf{e}(t_\beta^+) \rangle &= \beta. \end{aligned} \tag{S26}$$

From these definitions follows that the expected velocities immediately after the turning event can be expressed in terms of the expected velocities immediately before it (see [1] for details):

$$\begin{aligned}\langle v(0^+)\rangle &= \alpha\langle v(0^-)\rangle,\\ \langle v(t_\beta^+)\rangle &= \beta\langle v(t_\beta^-)\rangle.\end{aligned} \tag{S27}$$

On the other hand, to get the expected velocity at the end of run commencing at $t = 0$ and interrupting at $t = t_\beta$ we may take the integral

$$\langle v(t_\beta^-)\rangle = \int_0^\infty \langle v(t)p(t)\rangle dt. \tag{S28}$$

Substituting (S5) into (S4) and using (S11) yields

$$p(t) = \lambda_0 \left[1 - A|\nabla c| z(t-T)\right] e^{-\lambda_0 \int_0^t \left[1 - A|\nabla c| z(t'-T)\right] dt'}. \tag{S29}$$

As before, expanding the exponential and keeping only the linear term, we can obtain

$$p(t) = \lambda_0 e^{-\lambda_0 t}\left[1 - A|\nabla c| z(t-T) + \lambda_0 A|\nabla c| \int_0^t z(t'-T)dt'\right]. \tag{S30}$$

According to (S27), we obtain the expression for $\langle v(t_\beta^+)\rangle$

$$\langle v(t_\beta^+)\rangle = \beta\left[\frac{\langle v(0^+)\rangle\lambda_0}{\lambda_0 + 2D_r} - \lambda_0 A|\nabla c|(I_1^{(1)} - I_2^{(1)})\right]. \tag{S31}$$

where

$$I_1^{(1)} = \int_0^\infty e^{-\lambda_0 t}\langle v(t) z(t-T)\rangle dt, \tag{S32}$$

and

$$I_2^{(1)} = \lambda_0 \int_0^\infty \left(\int_{t'}^\infty e^{-\lambda_0 t}\langle v(t) z(t'-T)\rangle dt\right) dt'. \tag{S33}$$

Similar calculations for the second run give us the expression for the expected velocity immediately before the turning of type α at $t = t_\alpha + t_\beta$

$$\langle v(t_\alpha^-)\rangle = \frac{\langle v(t_\beta^+)\rangle\lambda_0}{\lambda_0 + 2D_r} - \lambda_0 A|\nabla c|(I_1^{(2)} - I_2^{(2)}). \tag{S34}$$

But, since the expected velocity before this α-turning does not change from that was for α-turning at $t = 0$, i.e. $\langle v(t_\alpha^-)\rangle = \langle v(0^-)\rangle$, and since $\langle v(0^+)\rangle = \alpha\langle v(0^-)\rangle$, the equation (S34) can be rewritten as

$$\langle v(0^+)\rangle = \alpha\left[\frac{\langle v(t_\beta^+)\rangle\lambda_0}{\lambda_0 + 2D_r} - \lambda_0 A|\nabla c|(I_1^{(2)} - I_2^{(2)})\right]. \tag{S35}$$

The equalities (S31) and (S35) give us a system of linear equations with variables $\langle v(0^+)\rangle$ and $\langle v(t_\beta^+)\rangle$ that provide expressions for the cell's velocities at the beginning of run after type α and type β turning events, respectively

$$\begin{aligned}\langle v(0^+)\rangle &= \frac{\alpha A|\nabla c|\lambda_0 (\lambda_0 + 2D_r)^2}{(\lambda_0 + 2D_r)^2 - \alpha\beta\lambda_0^2}\left[\frac{\beta\lambda_0}{\lambda_0 + 2D_r}\left(I_2^{(1)} - I_1^{(1)}\right) - I_1^{(2)} + I_2^{(2)}\right],\\ \langle v(t_\beta^+)\rangle &= \frac{\beta A|\nabla c|\lambda_0 (\lambda_0 + 2D_r)^2}{(\lambda_0 + 2D_r)^2 - \alpha\beta\lambda_0^2}\left[\frac{\alpha\lambda_0}{\lambda_0 + 2D_r}\left(I_2^{(2)} - I_1^{(2)}\right) - I_1^{(1)} + I_2^{(1)}\right].\end{aligned} \tag{S36}$$

Finally, we combine all preceding results according to (S1), and thus arrive at the chemotactic drift speed for the delta-response

$$v_\delta = \frac{v_0^2}{3}\lambda_0 A|\nabla c|\left[k_\delta \cosh\left(\sqrt{\alpha}\sqrt{\beta}\lambda_0 T\right) + m_\delta \sinh\left(\sqrt{\alpha}\sqrt{\beta}\lambda_0 T\right) + n_\delta\right] \tag{S37}$$

where denoting $s_{\alpha\beta} = \alpha + \beta$ and $d_{\alpha\beta} = -1 + \alpha\beta$ the coefficients k_δ, m_δ, n_δ are:

$$\begin{aligned}
k_\delta &= \frac{\lambda_0 e^{-(2D_r+\lambda_0)T}\left(4D_r^2\left(2 - s_{\alpha\beta}\right) - 8D_r d_{\alpha\beta}\lambda_0 - \left(2 + s_{\alpha\beta}\right) d_{\alpha\beta}\lambda_0^2\right)}{2\left(4D_r^2 + 4D_r\lambda_0 - d_{\alpha\beta}\lambda_0^2\right)^2}, \\
m_\delta &= \frac{\lambda_0 e^{-(2D_r+\lambda_0)T}\left(4D_r^2\left(s_{\alpha\beta} - 2\alpha\beta\right) - 4D_r d_{\alpha\beta}\lambda_0 s_{\alpha\beta} - \lambda_0^2 d_{\alpha\beta}\left(s_{\alpha\beta} + 2\alpha\beta\right)\right)}{2\left(4D_r^2 + 4D_r\lambda_0 - d_{\alpha\beta}\lambda_0^2\right)^2\sqrt{\alpha}\sqrt{\beta}}, \\
n_\delta &= \frac{\lambda_0^2\left(4D_r^2\left(-s_{\alpha\beta} + 2\alpha\beta\right) + 4D_r\lambda_0 d_{\alpha\beta}s_{\alpha\beta} + \lambda_0^2 d_{\alpha\beta}\left(s_{\alpha\beta} + 2\alpha\beta\right)\right)}{2\left(2D_r + \lambda_0\right)\left(4D_r^2 + 4D_r\lambda_0 - d_{\alpha\beta}\lambda_0^2\right)^2},
\end{aligned} \tag{S38}$$

Having found v_δ, we obtain the chemotaxis drift speed for the response function

$$R(T) = W\lambda_0 e^{-\lambda_0 T}\left[1 - \frac{\lambda_0 T}{2} - \left(\frac{\lambda_0 T}{2}\right)^2\right], \tag{S39}$$

where W is a single normalization constant with the dimension of volume, according to

$$v_d = \int_0^\infty R(T)\frac{v_\delta(t)}{A}dT, \tag{S40}$$

that yields

$$v_d = \frac{v_0^2\lambda_0^2 W|\nabla c|\sum_{j=0}^{7} a_j(\alpha,\beta)D_r^{7-j}\lambda_0^j}{4\sum_{j=0}^{10} b_j(\alpha,\beta)D_r^{10-j}\lambda_0^j}, \tag{S41}$$

where

$$\begin{aligned}
a_0(\alpha,\beta) &= -256\left(-2 + s_{\alpha\beta}\right), \\
a_1(\alpha,\beta) &= -64\left(-46 + 12\alpha\beta + 17s_{\alpha\beta}\right), \\
a_2(\alpha,\beta) &= -32\left(-222 + 116\alpha\beta + (51 + 2\alpha\beta)s_{\alpha\beta}\right), \\
a_3(\alpha,\beta) &= 16\left(582 - 448\alpha\beta + 20\alpha^2\beta^2 - (48 + 29\alpha\beta)s_{\alpha\beta}\right), \\
a_4(\alpha,\beta) &= 8\left(894 - 896\alpha\beta + 108\alpha^2\beta^2 + (63 - 126\alpha\beta + 10\alpha^2\beta^2)s_{\alpha\beta}\right), \\
a_5(\alpha,\beta) &= -4\left(-804 + 994\alpha\beta - 222\alpha^2\beta^2 + 4\alpha^3\beta^3 + (-183 + 250\alpha\beta - 53\alpha^2\beta^2)s_{\alpha\beta}\right), \\
a_6(\alpha,\beta) &= -2\left(-392 + 590\alpha\beta - 194\alpha^2\beta^2 - 4\alpha^3\beta^3 + (-152 + 243\alpha\beta - 97\alpha^2\beta^2 + 6\alpha^3\beta^3)s_{\alpha\beta}\right), \\
a_7(\alpha,\beta) &= -d_{\alpha\beta}^2\left(-80 - 12\alpha\beta + 4\alpha^2\beta^2 + (-44 + 7\alpha\beta)s_{\alpha\beta}\right),
\end{aligned}$$

and

$$
\begin{aligned}
&b_0(\alpha,\beta) = 1024,\\
&b_1(\alpha,\beta) = 8192,\\
&b_2(\alpha,\beta) = 29184 - 1280\alpha\beta,\\
&b_3(\alpha,\beta) = 60928 - 8192\alpha\beta,\\
&b_4(\alpha,\beta) = 82496 - 22784\alpha\beta + 640\alpha^2\beta^2,\\
&b_5(\alpha,\beta) = 75648 - 35968\alpha\beta + 3072\alpha^2\beta^2,\\
&b_6(\alpha,\beta) = 47552 - 35248\alpha\beta + 6144\alpha^2\beta^2 - 160\alpha^3\beta^3,\\
&b_7(\alpha,\beta) = 20224 - 21952\alpha\beta + 6560\alpha^2\beta^2 - 512\alpha^3\beta^3,\\
&b_8(\alpha,\beta) = 5568 - 8480\alpha\beta + 3948\alpha^2\beta^2 - 624\alpha^3\beta^3 + 20\alpha^4\beta^4,\\
&b_9(\alpha,\beta) = 896 - 1856\alpha\beta + 1272\alpha^2\beta^2 - 344\alpha^3\beta^3 + 32\alpha^4\beta^4,\\
&b_{10}(\alpha,\beta) = 64 - 176\alpha\beta + 172\alpha^2\beta^2 - 73\alpha^3\beta^3 + 14\alpha^4\beta^4 - \alpha^5\beta^5.
\end{aligned}
$$

If we assume, that all turning angles are equal ($\alpha = \beta$), we obtain the following expression

$$
v_d = W|\nabla c|v_0^2 \frac{\lambda_0^2(1-\beta)(4D_r + \lambda_0(5-2\beta))}{6(2D_r + \lambda_0 - \beta\lambda_0)(2D_r + (2-\beta)\lambda_0)^3}, \tag{S42}
$$

which agrees with formula (27) in [3]. If we assume, that the cell's motion pattern is "run-tumble-flick" ($\alpha = 0$), we get

$$
\begin{aligned}
v_d = {} & \frac{W|\nabla c|v_0^2\lambda_0^2}{192(D_r+\lambda_0)^4(2D_r+\lambda_0)^2}\cdot \\
& (16D_r^3(2-\beta) + 4D_r^2\lambda_0(22-5\beta) + 2D_r\lambda_0^2(38+5\beta) + \lambda_0^3(20+11\beta)),
\end{aligned} \tag{S43}
$$

which also agrees with formula (28) in [3].

References

1. Locsei JT. Persistence of direction increases the drift velocity of run and tumble chemotaxis. J Math Biol. 2007; 55:41–60.

2. de Gennes PG. Chemotaxis: the role of internal delays. Eur Biophys J. 2004; 33: 691–693.

3. Taktikos J, Stark H, Zaburdaev V. How the Motility Pattern of Bacteria Affects Their Dispersal and Chemotaxis. PLoS ONE. 2013; 8(12): e81936.
