# Supplementary material for: Chemotactic drift speed for bacterial motility pattern with two alternating turning events
Source: PLoS One. 2018 Jan 19;13(1):e0190434. doi: 10.1371/journal.pone.0190434 (PMC5774696; doi:10.1371/journal.pone.0190434)
Supplement: S2 Appendix — (PDF) [file pone.0190434.s002.pdf]

Chemotactic drift speed for bacterial motility pattern with two alternating turning events

Evgeniya V. Pankratova[1*], Alena I. Kalyakulina[1], Mikhail I. Krivonosov[1], Sergei V. Denisov[1, 2], Katja M. Taute[3], Vasily Yu. Zaburdaev[4, 5].

1 Institute of Information Technologies, Mathematics and Mechanics, Lobachevsky State University, Nizhniy Novgorod, Russia
2 Department of Theoretical Physics, University of Augsburg, Germany
3 Rowland Institute at Harvard, Harvard University, Cambridge, USA
4 Max Planck Institute for the Physics of Complex Systems, Dresden, Germany
5 Institute of Supercomputing Technologies, Lobachevsky State University, Nizhniy Novgorod, Russia

*E-mail: evgenia.pankratova@itmm.unn.ru

Supporting information

S2 Appendix. Simulation algorithm
To verify the obtained analytical results, we performed numerical sampling over an ensemble of chemotaxis trajectories. In this section we describe the used simulation algorithm. The algorithm consists of the following steps: (i) choosing a primary direction of movement, (ii) swimming in the selected direction, (iii) reorientation of bacteria with some probability.

We first consider the dynamics in between two consecutive reorientation events, i.e., steps (i) and (ii). Bacteria swim in a fluid medium and thus are subjected to thermal fluctuations. In addition there can be fluctuations of other origins, e.g. coming from molecular flagellar motors. In our model all these effects are adsorbed into the fluctuations of the cell's swimming direction. In terms of the cell velocity this is the standard rotational diffusion (since the absolute value of the velocity remains constant) and thus the process can be parametrized with two angles of the spherical coordinate system, θ and φ. Coordinates of the cell are then obtained by integrating the velocity vector in time.

Langevin dynamics of the angles is described by a pair of stochastic equations,

$$\dot{\theta} = D_r \cot\theta + \sqrt{2D_r}\xi_\theta(t), \quad \dot{\varphi} = \frac{\sqrt{2D_r}}{\sin\theta}\xi_\varphi(t), \tag{1}$$

where $\xi_\theta(t)$ and $\xi_\varphi(t)$ are two independent standard Gaussian random variables of dispersion one, and D_r is the rotational diffusion coefficient.

These stochastic equations results in a Fokker-Planck equation for the probability density $P(\theta, \varphi, t)$:

$$\frac{\partial P}{\partial t} = D_r \nabla P = D_r \left[\frac{1}{\sin\theta}\frac{\partial}{\partial\theta}(\sin\theta\frac{\partial}{\partial\theta}) + \frac{1}{\sin^2\theta}\frac{\partial^2}{\partial\varphi^2} \right] P. \tag{2}$$

More practically, if the time propagation step is short, the new direction of the velocity vector (with respect to the initial direction) is given by $\phi \simeq \arccos(1 - 2D_r\Delta t)$. A new vector should be chosen randomly on the corresponding cone.

Next we consider a reorientation event, step (iii). In an environment with a chemoattractant gradient, the run intervals are no longer isotropic in space; they depend (statistically) on the direction of the motion. They change differently depending

on whether the bacterium moves up or down the gradient. The statistics (rate) of changes is determined, according to the linear theory [1], by Eq. (1). The specific form of the memory kernel, Eq. (2), allows to transform the original non-Markovian (non-local in time) dynamics into a local one by extending the dimension of the space of dynamical variables. This technique is well-known in the field of stochastic processes driven by colored noise where it is called "embedding"; see, e.g., Refs. [2, 3].

Avoiding technical details, we present the final results [4]. The variation of the reorientation rate is given by

$$\Delta(t) = \int_{-\infty}^{t} R(t-\tau)c(\tau)d\tau = m_0(t) - \frac{m_1(t)}{2} - \frac{m_2(t)}{4}, \quad (3)$$

where three additional variables m_i are determined form a system of coupled linear differential equations,

$$\begin{cases} \dot{m_0} = -\lambda_0 m_0 + c(t), \\ \dot{m_1} = -\lambda_0 m_1 + m_0, \\ \dot{m_2} = -\lambda_0 m_2 + m_1. \end{cases} \quad (4)$$

where $c(t) = c(\boldsymbol{r}(t))$ is the chemical concentration, measured at the location of the bacterium $\boldsymbol{r}(t)$ at the time t.

Now we are ready to describe the numerical algorithm. After every integration step, the bacterium re-orients itself in a new random direction with the probability $p = \min(1, \lambda(t)\Delta t)$, where Δt is the length of single integration step, or, alternatively, it continues to move in the same direction with probability $1 - p$. Finally, the algorithm is a sequence of the following steps:

1. Calculate reorientation frequency $\lambda(t)$ for the current state of the bacteria using linear chemotaxis theory. For this, solve the system of equations (4), e.g., by using Euler's method with small step Δt. Reorientation of bacteria occurs with probability $p = \min(1, \lambda(t)\Delta_r t)$ according to the pattern. Thereby, calculate the new bacteria speed vector.

2. Calculate the new bacteria position at the time Δt (by using the standard linear Euler scheme).

3. For applying the rotational diffusion, calculate a new speed vector, i.e. rotate it by an angle $\phi = \arccos(1 - 2D\Delta t)$. It gives a cone of directions, obtained by such rotation. We need to choose one of the directions on the cone, which we do by choosing on a unit circle (the base of the cone) a point following the uniform distribution. Thus we define a new velocity vector.

References

1. Block SM, Segall JE, Berg HC. Adaptation kinetics in bacterial chemotaxis. J Bacteriol. 1983; 154:312–323.

2. Grabert H, Talkner P, Hänggi P. Microdynamics and Time-Evolution of Macroscopic Non-Markovian Systems. Z Physik B. 1977; 26, 389.

3. Kupferman R. Fractional kinetics in Kac-Zwanzig heat bath models. J Stat Phys. 2004; 114, 291.

4. Celani A, Vergassola M. Bacterial strategies for chemotaxis response. Proc Natl Acad Sci USA. 2009; 107: 1391-1396.
